# Aquatic Invertebrates as Unlikely Vectors of Buruli Ulcer Disease

**DOI:** 10.3201/eid1408.071503

**Published:** 2008-08

**Authors:** M. Eric Benbow, Heather Williamson, Ryan Kimbirauskas, Mollie D. McIntosh, Rebecca Kolar, Charles Quaye, Felix Akpabey, D. Boakye, Pam Small, Richard W. Merritt

**Affiliations:** * Michigan State University, East Lansing, Michigan, USA; † University of Tennessee, Knoxville, Tennessee, USA; ‡ University of Ghana, East Legon, Ghana; § Water Resources Research Institute, Accra, Ghana; 1 Current affiliation: University of Dayton, Dayton, Ohio, USA.

**Keywords:** Buruli ulcer, *Mycobacterium ulcerans* infection, disease vectors, macroinvertebrates, transmission, environmental reservoirs, research

## Abstract

Buruli ulcer is a necrotizing skin disease caused by *Mycobacterium ulcerans* and associated with exposure to aquatic habitats. To assess possible transmission of *M. ulcerans* by aquatic biting insects, we conducted a field examination of biting water bugs (Hemiptera: Naucoridae, Belostomatidae, Nepidae) in 15 disease-endemic and 12 non–disease-endemic areas of Ghana, Africa. From collections of 22,832 invertebrates, we compared composition, abundance, and associated *M. ulcerans* positivity among sites. Biting hemipterans were rare and represented a small percentage (usually <2%) of invertebrate communities. No significant differences were found in hemipteran abundance or pathogen positivity between disease-endemic and non–disease-endemic sites, and between abundance of biting hemipterans and *M. ulcerans* positivity. Therefore, although infection through insect bites is possible, little field evidence supports the assumption that biting hemipterans are primary vectors of *M. ulcerans*.

## Abstract 2

Biting water bugs were not correlated with pathogen occurrence.

*Mycobacterium ulcerans* infection is an emerging skin disease often called Buruli ulcer (BU). Infection results in illness and lasting negative socioeconomic effects in rural areas of the tropics and subtropics (*1*). The pathologic changes, clinical signs and symptoms, and treatment have been reviewed elsewhere (*2*–*5*). In this article we evaluate field evidence for the potential of aquatic invertebrates to be vectors of *M. ulcerans*.

The exact mode of BU transmission remains unknown; however, past epidemiologic studies have associated BU with human activity near, or within, slow-flowing or standing water bodies that have been created or disturbed by humans (*2*–*4*). Although several water-related risk factors have been recognized, none has been consistently reported, making it difficult to identify specific water-related risk activities (*6*–*8*). Most studies suggest that infection occurs through inoculation of *M. ulcerans* into skin lesions or insect bites (*2*,*4*,*9*–*11*). Portaels et al. (*11*) were the first to propose that aquatic insects might serve as vectors of *M. ulcerans*. This hypothesis maintains that *M. ulcerans* is found in biofilms of aquatic habitats and concentrated by grazing or filter-feeding invertebrates that are then consumed by predators known to bite humans (*11*). Initial evidence for this hypothesis used PCR detection of the insertion sequence IS*2404* to document *M. ulcerans’* association with biting water bugs (Hemiptera), filtered concentrates of water, detritus, and aquatic plants (*4*,*12*–*14*). These studies were important for understanding the possible environmental reservoirs of *M. ulcerans*. However, IS*2404* is now understood to be not specific for *M. ulcerans* because this insertion sequence has been found in a number of other aquatic mycobacterial species, including *M. marinum* (*15*–*17*). When more discriminatory methods based on detection of variable number tandem repeats were used, many IS*2404-*positive environmental samples were reported to lack *M. ulcerans* (*18*). In light of these recent findings, the relative frequency or abundance of *M. ulcerans* among aquatic invertebrates or other environmental reservoirs, remains tenuous, and thus, the role of aquatic insect vectors is uncertain.

A series of laboratory experiments provided initial evidence for biting hemipteran vectors of *M. ulcerans* (*19*–*23*). Marsollier et al. (*9*,*24*) demonstrated that a South American isolate of *M. ulcerans* could survive and multiply within the salivary glands of aquatic bugs indigenous to France (Naucoridae: *Naucoris* *cimicoides*). Furthermore, *N. cimicoides* could transmit *M. ulcerans* by feeding on inoculated prey and then biting mice, which then exhibited BU (*9*). Most recently, exposure to hemipteran insect saliva was reported to infer protection against lesion development in laboratory mouse models (*21*). That study also reported correlations between aquatic insect salivary gland antibodies in humans categorized as exposed or patient, when the former group had exhibited BU. However, 3 limitations of that study have been noted (*25*): 1) the antibodies against salivary proteins might only be biomarkers of protection; 2) possible geographically related polymorphisms in the salivary proteins among hemipteran taxa could limit the generalizability of protection among distant communities; and 3) the overall relevance of biting aquatic insects infected with *M. ulcerans* in the natural environment is unknown.

A confounding factor in these experimental studies is that they used 1 South American isolate of *M. ulcerans.* Recent data support 2 major lineages of *M. ulcerans:* the ancestral strains that closely resemble *M. marinum* in chromosomal content, and the classic strains that have undergone substantial genome reduction (*26*). The latter strains account for all severe disease and include the African, Malaysian, and Australian isolates. The aforementioned laboratory studies have been elegantly performed, but the use of a French species of Naucoridae and a South American isolate of *M. ulcerans* makes it difficult to assess the importance of insect transmission in Africa. Thus, although provocative experimental data support a potential role for aquatic hemipterans as vectors of *M. ulcerans* in laboratory settings, no supporting evidence has been obtained from studies conducted in the natural setting. Results from field studies that identify the relative abundance and exposure potential of biting aquatic hemipterans can provide insight into the importance of biting insects in BU transmission.

This study had 3 objectives: 1) to describe the aquatic invertebrate samples collected during a large-scale, 2-year standardized field-sampling program of 27 bodies of water in Ghana, West Africa; 2) to investigate *M. ulcerans* positivity among the same aquatic invertebrates from those water bodies, directly linking aquatic invertebrate communities with pathogen positivity; and 3) to discuss the role of human-biting hemipterans as primary vectors of *M. ulcerans*. Data on the detection of *M. ulcerans* within aquatic samples based on the use of variable number tandem repeats analysis are presented in another article (*18*). In the current article, we associate presumptive *M. ulcerans* positivity rates with relative abundance and percentage composition of the same aquatic communities.

## Methods

### Study Sites

**Figure 1:**
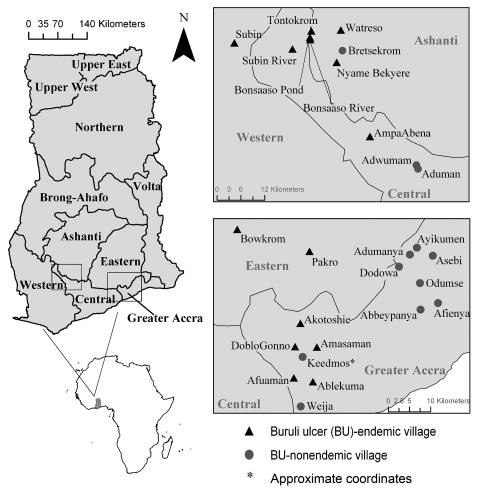
Regional site map of water bodies sampled in Ghana for aquatic invertebrates during 2004, 2005, or both. Small maps on left show location of Ghana in Africa and location of regions sampled within Ghana (boxes).

In June 2004 and August 2005, we sampled 27 water bodies associated with human communities in southern Ghana (Figure 1). The water bodies were located within or very near (<100–200 m) each community of housing structures and were routinely used for daily domestic purposes and reflect habitats of routine human exposure. These water bodies were chosen after discussions with community members who directed us to the main water source for drinking water, recreation, domestic washing, irrigation, or bathing for that community. Six of these sites were sampled in both years, providing information on annual variation: Afuaman, Amasaman, Abbeypanya, Afienya, Odumse, and Weija. Human BU case data for the years 2003–2005 were provided by the Ghana Ministry of Health and used to classify communities into 2 site types: 15 BU–endemic (BU+) and 12 BU–nonendemic (BU–). A site was classified as a BU+ type if at least 1 case of BU had been reported during the 3-year period.

### Aquatic Invertebrate Sampling and Processing

Within each water body, two 10–20-m transects were measured parallel to the shoreline and positioned through the dominant macrophyte community. Along each transect, we randomly placed two 1-m^2^ polyvinyl chloride quadrats and collected invertebrates by sweeping within the quadrat with a 500-µm mesh dip net. The quadrats floated on top of the water and delineated 1 m^2^ of area to be sampled by using an aquatic dip net designed to capture the aquatic life stages of invertebrates. Three sweeps of the dip net were performed from the water surface to the bottom substrate for comprehensive sampling of specimens in the water column. All contents were washed through a 500-µm sieve and preserved in 100% ethanol for laboratory identification and PCR. The 2 quadrats were combined into 1 composite sample.

### *M. ulcerans* Detection in Invertebrate Samples

Samples were analyzed in a 2-step procedure so that an initial screening reduced sample numbers. Small invertebrates were analyzed in pools of 3–15, whereas larger specimens were tested individually. DNA was extracted by using a protocol adapted from Lamour and Finley (*27*). Samples were ground and vortexed in 400 μL of lysis solution (100 mmol/L Tris, pH 8.0), 50 mmol/L EDTA, 500 mmol/L NaCl, 1.33% sodium dodecyl sulfate, and 0.2 mg/mL RNase A) and 1 g of 1.0-mm glass beads (Sigma-Aldrich, St. Louis, MO, USA), then centrifuged. After 150 μL of 5 mol/L potassium acetate was added, each sample was incubated overnight at –20° C. After a 30-min centrifugation, supernatants were transferred to new tubes containing 0.66 mol/L guanidine hydrochloride in a 63.3% ethanol solution. The samples were added to a spin filter (MO BIO Laboratories Inc., Carlsbad, CA, USA) in a 2-mL microcentrifuge tube (MO BIO Laboratories Inc.). The flow-through was discarded and the filter was rinsed first with 500 μL of wash solution (10 mmol/L Tris, pH 8, 1 mmol/L EDTA, 50 mmol/L NaCl, 67% ethanol) and then with 500 μL of 95% ethanol. The spin filters were dried by centrifugation and transferred to new 2-mL microcentrifuge tubes, immersed in 200 μL elution solution (10 mmol/L Tris, pH 8), and incubated at room temperature for 15 min. The DNA was eluted and stored at –20°C.

Presumptive identification of *M. ulcerans* in invertebrates was based on detection of the enoyl reduction domain (ER) in *mlsA* that encodes the lactone core of the mycolactone toxin, the major virulence determinant of *M. ulcerans.* All samples were screened for the presence of the ER gene, which has been evaluated for *M. ulcerans* specificity in a companion study that used a multitiered PCR approach (*18*). Amplification of the ER gene was achieved using a 50-μL reaction mixture containing 1 μL each of forward and reverse primer (*15*,*18*), 10 μL 5× Go Taq reaction buffer (Promega, Madison, WI, USA), 1 μL 10 mmol/L PCR nucleotide mix (Promega), 31.7 μL double-distilled water, 1.6 units Go Taq polymerase enzyme (Promega), and 5 μL DNA template. Cycling conditions began with an initial denaturation at 94°C for 5 min, 35 cycles of 94°C for 1 min, 58°C for 45 seconds, 72°C for 1 min, and a final 10-min extension at 72°C. The amplified DNA was subjected to gel electrophoresis by using a 1.5% agarose gel, and band sizes were compared by using a 1-kb DNA ladder (Invitrogen, Carlsbad, CA, USA). PCR products of appropriate size were cloned into the pCR2.1 Topo vector (Invitrogen) and sequenced by using an ABI 3100 automated genetic analyzer (Applied Biosystems, Foster City, CA, USA).

### Data Analysis

Using all invertebrate data, we initially evaluated differences between site types (i.e., BU+ vs BU–) by comparing total abundance and percentage composition. Only those taxa that represented >3% of total invertebrates collected from all sites were used for subsequent statistical analyses because some taxa were so rare that any comparisons would limit meaningful conclusions. However, because we were interested in evaluating Hemiptera known to bite humans, the families Belostomatidae, Naucoridae, and Nepidae also were included, although each represented <2% of total collections.

To compare abundance differences between site types, *t* tests were used after data were log + 1 transformed to meet the assumptions of normality and equal variances. For percentage composition differences, data were arc-sine square root transformed, but they still did not demonstrate a normal distribution, so the nonparametric Wilcoxon/Kruskal-Wallis rank sum test was used. Because multiple tests were performed, it was necessary to calculate a Bonferroni adjusted α (and corresponding p value) of 0.006 to assist in interpreting statistically significant differences. However, to evaluate the biological meaning of these multiple tests, Cohen *d* effect size (and 95% confidence intervals) was calculated with Hedges adjustment (*28*). To compare overall ER positivity proportions between BU+ and BU– sites, a *t* test was used after data were arc-sine square root transformed. Lastly, we evaluated correlations between total biting hemipterans (and each individual family) and ER positivity using Spearman rank correlations with a Bonferroni adjusted α = 0.008. This nonparametric test was used after attempts to transform the data for normality and homogeneity of variances failed.

## Results

### Invertebrate Abundance and Composition

Of 22,832 invertebrates collected, ≈50% came from each group of BU+ and BU– site types (Technical Appendix). A total of 85 taxa were represented among all sites: 80 taxa were collected from BU+ sites compared with 71 from BU– sites. The abundance of specific taxa was not consistent between site types, indicating that the invertebrate communities were highly variable. This variability was confirmed in statistical analyses comparing the most abundant taxa (>3%) with substantial effect size variation within and among taxa (Technical Appendix). The invertebrates found in greatest abundance were 2 families of Diptera (i.e., Chironomidae and Culicidae), 1 family of Ephemeroptera (Baetidae), and several Crustacea. More than 300 individuals of some families of Hemiptera, Coleoptera, and Odonata were encountered (Technical Appendix). The biting Hemiptera were usually rare. For instance, 55 Naucoridae in total were collected, which was about 0.2% of all invertebrates sampled (Technical Appendix).

**Figure 2:**
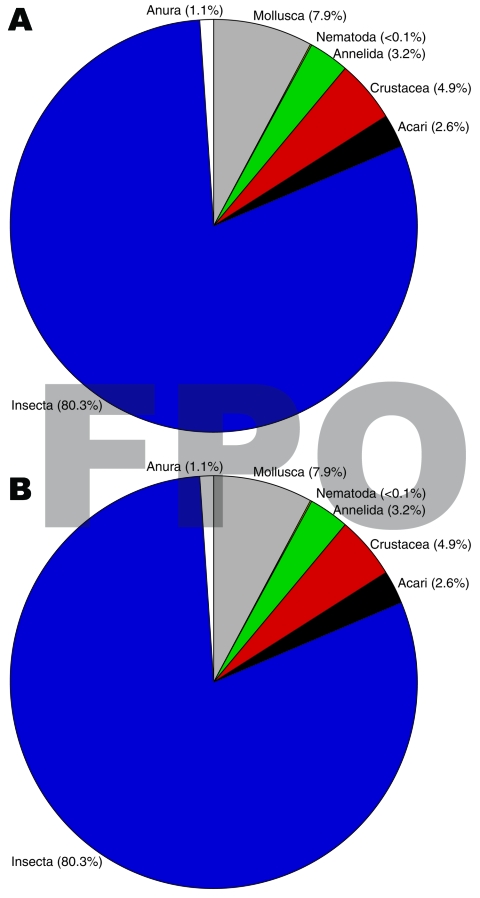
Higher level classification (e.g., class, phylum) taxa percentage composition between A) Buruli ulcer–endemic (n = 15) and B) Buruli ulcer–nonendemic (n = 12) site types, Ghana.

**Figure 3:**
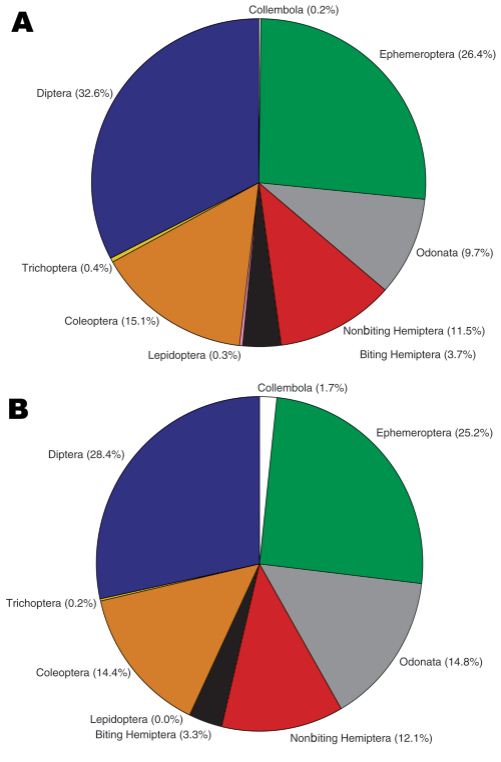
Insect order percentage composition between Buruli ulcer–endemic (n = 15) and Buruli ulcer–nonendemic site types (n = 12), Ghana.

Insects made up the greatest percentage of the invertebrates collected from BU+ sites but were nearly equivalent to the Crustacea in BU– sites. In BU– sites, Anura made up a relatively higher percentage, but most (1,231 of 1,303 individuals) were from a single site (Figure 2; Technical Appendix). The Crustacea were most often represented by copepods, ostracods, and shrimp (Atyidae); fewer shrimp were collected from BU+ sites. Most shrimp were from BU– sites Adumanya (197) and Keedmos (120). Further, in BU– sites the large copepod abundance occurred primarily at Odumse, where 1,723 were collected from a total 1,884 (Technical Appendix). Insects were reduced by 40% in BU– sites compared with BU+ sites (Figures 2, 3). When individual insect orders were compared, the Ephemeroptera (mayflies) and Diptera (true flies) made up the greatest percentages of insects in both BU+ and BU– site types (Figure 3; Technical Appendix).

When the abundance and percent composition of dominant taxa were statistically compared between BU+ and BU– site types, there were no significant differences for any taxa (Technical Appendix). However, the effect size varied greatly, reflecting a need to collect from more sites in future studies. On average, the Chironomidae (midges) made up the greatest percentage of the invertebrate communities, representing 9%–20% of the total, while the Baetidae (mayflies) ranged from 6% to 15% and the Culicidae (mosquitoes) from 2% to 5%. The biting Hemiptera made up a very small percentage of the dominant invertebrate communities, with Naucoridae <0.5%, Belostomatidae <2%, and Nepidae <0.3% (Technical Appendix).

### Presumptive Identification of *M. ulcerans* from Invertebrates

**Figure 4:**
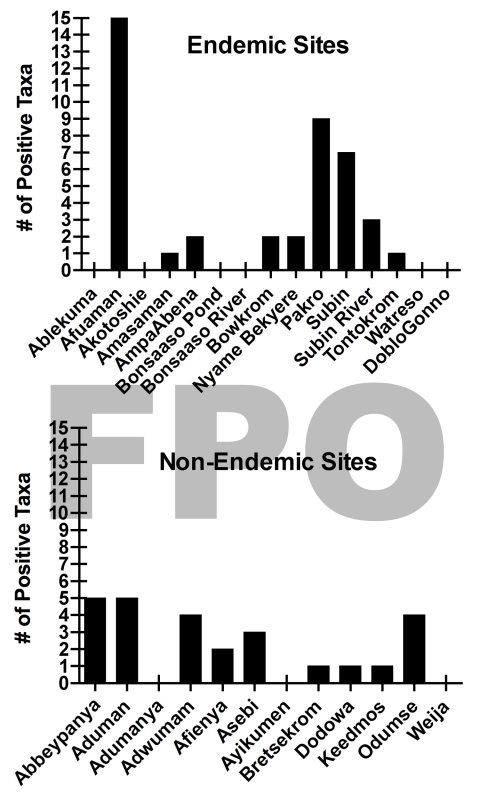
Number of enoyl-reduction-domain–positive taxa detected for each A) Buruli ulcer–endemic site (n = 15) and B) Buruli ulcer–nonendemic site (n = 12), Ghana.

Presumptive identification of *M. ulcerans* from a total of 1,032 invertebrate sample pools tested found no significant difference between BU+ and BU– site types (Technical Appendix). Furthermore, there was no detectable pattern of invertebrate taxa ER positivity among sites, indicating that no single taxon was more often likely to be positive at a particular site. The number of ER positive taxa that were detected at any site ranged from 0 to 15 and 0 to 6 in BU+ and BU– sites, respectively (Figure 4). Clearly, not all BU+ or BU– sites had ER positive invertebrates. There were 6/15 BU+ sites without a single taxon positive compared with only 3/12 BU– sites (Figure 4).

Taxon-specific ER positivity was highly variable, and percentage positivity ranged from 0% to 100% among taxa (Technical Appendix). There were 26 taxa positive from BU+ site types compared with only 18 from BU– sites. Only 2 taxa were positive in BU– and not in BU+ sites, and for those taxa, <5 samples were tested from the BU+ type. When only those taxa with >5 samples tested were compared, no observable pattern in ER positivity was apparent among sites or taxa. The most abundant taxa did not always have the greatest ER positivity. For instance, positivity of Chironomidae (19.5% of all invertebrates) was only about 7%, even though positivity of Caenidae (<2% of all invertebrates) ranged from 6% to 17% (Technical Appendix). For taxa with >5samples tested from either BU+ or BU– sites, the ER positivity was >20% for 5 taxa and from 10% to 20% for 12 taxa (Technical Appendix). The biting Hemiptera had neither the highest nor consistently higher ER positivity compared with more abundant taxa (Technical Appendix). Fifteen taxa with >5 samples tested had 0 positivity. These taxa represented all invertebrate functional feeding groups (e.g., predators, shredders, scrapers, collector-gatherers, and filterers).

### Biting Hemiptera Correlations

No significant correlation was found between mean ER positivity and total biting Hemiptera (r = 0.25; p = 0.218) or any individual family: Belostomatidae (r = 0.31; p = 0.118), Naucoridae (r = -0.03; p = 0.850), and Nepidae (r = 0.37; p = 0.060). These results confirmed that biting Hemiptera were not significantly associated with the pathogen in the environment.

## Discussion

The role of aquatic invertebrates in the transmission of BU has been proposed several times (*3*,*4*,*29*). However, to date, no large-scale field studies have assessed aquatic invertebrate communities from multiple locations or evaluated associated *M. ulcerans* positivity rates for specific invertebrate communities. Understanding the relative abundance and composition of the invertebrate taxa is a useful initial approach for assessing exposure risk of populations that use waterbodies for domestic needs. If biting water bugs are primary vectors of *M. ulcerans*, then the minimum (but not only) supporting evidence should confirm at least 1 of the following characteristics: 1) biting water bugs should be relatively more abundant at sites with BU cases compared with those without BU, indicating increased exposure potential to the vector in disease-endemic communities; 2) biting water bugs should have relatively higher *M. ulcerans* positivity rates within disease-endemic sites compared with disease-nonendemic sites; 3) *M. ulcerans* positivity rates should be higher in biting water bugs than in other invertebrates in the same sites, demonstrating increased potential pathogen exposure in the vector compared with background exposure; or 4) a correlation should exist between *M. ulcerans* positivity and vector abundance. This study addressed each of these characteristics and did not find strong confirming evidence that biting water bugs were any more important in the transmission of *M. ulcerans* than passive contact exposure to the environment. This finding is consistent with reports that few infected persons remember being bitten by water bugs (*30*). Although our results do not prove that infection could never occur from biting water bugs, they suggest that such an event would be rare.

In a companion study, Williamson et al. (*18*) reported *M. ulcerans* ER positivity from a broad spectrum of environmental samples, including animals, water filtrate, and biofilm on glass slides. They found that *M. ulcerans* DNA was detectable, not only at sites with or without a history of BU cases, but also in the environment, independent of invertebrates; positive results were detected for all sample types. Although *M. ulcerans* has been detected on the exoskeleston of experimentally infected Naucoridae (*9*), the possibility that invertebrates could serve as substrates for *M. ulcerans* in a natural environment has not been addressed, but it is certainly possible and may explain the wide range of taxa that were found positive in this study.

The invertebrate communities in this study demonstrated high intersite variation (Technical Appendix), a finding similar to those of other studies of lentic invertebrate habitats (*31*,*32*). This variation suggests that additional collection sites should be included for a more comprehensive evaluation of invertebrate communities; an expanded study is under way. Hydrologic and physical/chemical attributes regulate the structure and abundance of invertebrate communities (*31*), while biotic factors such as macrophytes and fish can also influence communities (*33*). Few basic ecologic studies have been conducted on non–disease-related aquatic invertebrates in West Africa. The most comprehensive articles on ecology have come from studies of small, fast-flowing streams or large lakes (*34*,*35*), which are different habitats than those in this study.

Season may also play a role in invertebrate abundance patterns; however, in many tropical and subtropical regions, most invertebrate taxa show minimal seasonally based abundance patterns (*36*–*38*). Most tropical species have multivoltine (multiple generations) and asynchronous (overlapping) life cycles throughout the year (*39*). For instance, all life stages of tropical naucorids have been reported through both wet and dry seasons over 2 years (*38*), and the same has been documented for other aquatic invertebrates in Kenya (*36*) and Lake Tanganyika (*37*). Therefore, although season might have had a small effect on the abundance variation of biting hemipterans and other invertebrates, this influence was unlikely to have limited our potential for detecting differences between BU+ and BU– sites.

If season affects biting Hemiptera populations, and these insects are important vectors, then human BU case data should reflect seasonal patterns, but this is not generally reported (*4*). In a recent study, no seasonal pattern was shown in monthly BU cases for 2003, 2004, and 2005 (*40*). In the current study, sampling each site throughout the year was not logistically feasible. In other ongoing studies, we have sampled an additional 55 sites, including 22 sites from 2004 to 2005 that have been sampled at least twice and 6 sites sampled 3 times over 3 years. The abundance of biting Hemiptera and other invertebrates from these additional sites are similar to what is reported here. Therefore, although season may have influenced our invertebrate community abundances, little evidence suggests that BU+ and BU– sites would be differentially affected, that Ghanaian invertebrate communities should respond differently to season compared with communities in other tropical and subtropical regions, or that any seasonal pattern in BU cases is related to seasonal population changes of biting hemipterans.

Various researchers have proposed that biting water bugs could be vectors for *M. ulcerans*, and laboratory studies have provided evidence for this possibility. However, no complementary field studies had tested these laboratory results. Results from this field study do not support the hypothesis that biting aquatic insects are primary vectors of *M. ulcerans*. The results do not rule out the possibility of biting Hemiptera or other invertebrates as vectors or possible reservoirs for *M. ulcerans*, but rather, they suggest caution in describing their role in transmission. These field data on biting hemipteran abundance and *M. ulcerans* positivity suggest a need to reevaluate future research directions for understanding BU transmission.

## Supplementary Material

Technical Appendix Aquatic Invertebrates as Unlikely Vectors of Buruli Ulcer Disease
