# Supplementary material for: Aquatic Invertebrates as Unlikely Vectors of Buruli Ulcer Disease
Source: Emerg Infect Dis. 2008 Aug;14(8):1247–54. doi: 10.3201/eid1408.071503 (PMC2600397; doi:10.3201/eid1408.071503)
Supplement: Technical Appendix — Aquatic Invertebrates as Unlikely Vectors of Buruli Ulcer Disease [file 07-1503_Techapp-s1.pdf]

Aquatic Invertebrates as Unlikely Vectors of Buruli Ulcer Disease

Technical Appendix

Table 1. Total number of individual organisms collected for each taxon and grouped as BU–endemic or BU–nonendemic, based on human BU case data from 2003–2005*

Higher classification	Lower classification	Family	No. BU endemic (n = 15)	No. BU nonendemic (n = 12)	Total
Bivalvia		Sphaeriidae	1	7	8
	Gastropoda	Ancylidae	22	37	59
		Bulininae	162	14	176
		Lymnaeidae	12	0	12
		Physidae	214	12	226
		Planorbidae	315	210	525
		Thiaridae	16	44	60
		Viviparidae	2	1	3
		Gastropoda	171	99	270
Nematoda			10	7	17
Oligochaeta			317	947	1,264
Hirudinae			55	34	89
Crustacea	Cladocera		164	605	769
	Mysidacea		0	2	2
	Conchostraca		6	0	6
	Copepoda		78	1,884	1,962
	Ostracoda		248	833	1,081
	Decapoda	Atyidae	74	450	524
Arachnida	Acari		299	409	708
Insecta	Collembola	Entomobryiidae	22	45	67
		Isotomidae	0	27	27
	Ephemeroptera	Baetidae	2,127	736	2,863
		Caenidae	312	305	617
		Heptageniidae	12	29	41
		Leptophlebiidae	11	4	15
		Oligoneuriidae	1	0	1
		Polymitarcyidae	1	10	11
	Odonata	Calopterygidae	0	2	2
		Coenagrionidae	385	283	668
		Corduliidae	94	73	167
		Gomphidae	1	1	2
		Libellulidae	131	107	238
		Unknown	66	30	96
		Protoneuridae	232	140	372
	Hemiptera	Belostomatidae	283	118	401
		Corixidae	47	217	264
		Gerridae	22	18	40
		Hebridae	10	0	10
		Hydrometridae	4	4	8
		Mesoveliidae	113	47	160
		Naucoridae	52	3	55
		Nepidae	12	22	34
		Notonectidae	198	189	387
		Ochteridae	1	0	1

		Pleidae	652	31	683
		Saldidae	2	1	3
		Veliidae	27	16	43
	Lepidoptera	Pyralidae	31	1	32
	Coleoptera	Carabidae	2	1	3
		Coleoptera	4	0	4
		Curculionidae	1	10	11
		Dryopidae	6	3	9
		Dytiscidae	272	86	358
		Elmidae	43	15	58
		Gyrinidae	23	2	25
		Hydraenidae	339	93	432
		Hydrophilidae	421	243	664
		Hygrobiidae	13	0	13
		Lampyridae	12	2	14
		Noteridae	251	83	334
		Psephenidae	1	0	1
		Scirtidae	52	44	96
		Staphylinidae	5	4	9
	Trichoptera	Ecnomidae	1	0	1
		Helicopsychidae	0	2	2
		Hydropsychidae	12	0	12
		Leptoceridae	18	6	24
		Polycentropodidae	3	0	3
	Diptera	Ceratopogonidae	126	135	261
		Chaoboridae	18	18	36
		Chironomidae	1,935	858	2,793
		Culicidae	871	182	1,053
		Diptera	1	1	2
		Empididae	6	0	6
		Ephydridae	9	3	12
		Muscidae	1	0	1
		Psychodidae	22	5	27
		Sciomyzidae	2	1	3
		Simuliidae	5	0	5
		Stratiomyiidae	15	9	24
		Syrphidae	3	1	4
		Tabanidae	13	1	14
		Thaumaleidae	0	2	2
		Tipulidae	15	6	21
Vertebrata	Anura		123	1,303	1,426
		Total	11,659	11,173	22,832

*BU, Buruli ulcer. Invertebrates were collected from sites during 2004, 2005, or both; all taxa that were collected are included.

Table 2. Mean (SD) invertebrate taxon abundance and percentage composition for taxa that made up >3% of the total collected, and calculated by sites grouped as BU endemic or BU nonendemic, based on human BU case data from 2003 through 2005*

		BU endemic (N = 15)		BU nonendemic (N = 12)			
Category	Taxon	Mean	SD	Mean	SD	p value	Cohen *d* (95% CI)
Abundance†	Total organisms	787	644	943	1,096	0.678	0.17 (–0.59–0.93)
	Ephemeroptera						
	Baetidae	142	257	61	82	0.241	0.41 (–0.36–1.18)
	Diptera						
	Culicidae	58	81	15	28	0.455	0.69 (–0.09–1.47)
	Chironomidae	129	106	72	108	0.113	0.52 (–0.25–1.29)
	Hemiptera						
	Belostomatidae	19	30	10	11	0.882	0.39 (–0.38–1.15)
	Naucoridae	3	6	0	1	0.050	0.68 (–0.10–1.46)
	Nepidae	1	1	2	4	0.768	0.33 (–0.43–1.10)
	Pleidae	43	71	3	5	0.066	0.77 (–0.02–1.56)
	Oligochaeta	23	30	73	188	0.787	0.36 (–0.41–1.13)
	Copepoda	5	11	157	494	0.587	0.42 (–0.35–1.19)
	Ostracoda	17	31	69	165	0.344	0.42 (–0.34–1.19)
	Cladocera	11	17	50	115	0.768	0.46 (–0.31–1.23)
	Acari	20	35	34	62	0.552	0.27 (–0.49–1.03)
	Anura	8	7	109	354	0.181	0.39 (–0.38–1.16)
% Composition‡	Ephemeroptera						
	Baetidae	15.1	16.1	6.1	4.6	0.053	0.71 (–0.07–1.50)
	Diptera						
	Culicidae	4.8	6.5	1.2	1.7	0.055	0.74 (–0.05–1.52)
	Chironomidae	19.5	19.2	8.8	7.8	0.065	0.71 (–0.08–1.49
	Hemiptera						
	Belostomatidae	1.4	1.9	2.1	3.6	0.544	0.24 (–0.52–1.00)
	Naucoridae	0.5	1.2	0.1	0.1	0.151	0.54 (–0.23–1.31)
	Nepidae	0.2	0.4	0.3	0.7	0.524	0.25 (–0.51–1.01)
	Pleidae	3.7	5.4	0.2	0.3	0.022	0.91 (0.11–1.70)
	Oligochaeta	2.2	2.7	3.5	5.4	0.476	0.28 (–0.48–1.05)
	Copepoda	0.6	1.2	5.3	13.8	0.246	0.48 (–0.29–1.25)
	Ostracoda	2.5	6.3	2.6	4.8	0.928	0.03 (–0.73– 0.79)
	Cladocera	1.4	2.1	3.1	5.7	0.369	0.36 (–0.40–1.13)
	Acari	1.7	2.1	4.8	11.6	0.392	0.35 (–0.41–1.12)
	Anura	1.5	1.9	7.1	17.8	0.309	0.42 (–0.35–1.19)

*BU, Buruli ulcer. Invertebrates were collected from sites during 2004, 2005, or both.
†Wilcoxon/Kruskal-Wallis rank sum test used for abundance comparisons.
‡*t* tests were used for percentage composition comparisons after data transformation. The effect size is given as Cohen *d* (with the Hedge adjustment) along with 95% confidence interval [CI]). Bonferroni multiple comparison adjustments gave an α = 0.006 for significant differences based on multiple comparisons.

Table 3. Community-specific ER PCR sample numbers and positivity results*

Community	No. ER negative	No. ER positive	No. samples tested (% ER positive)
Endemic			
Ablekuma	23	0	23 (0)
Afuaman	67	17	84 (20.2)
Akotoshie	27	0	27 (0)
Amasaman	58	1	59 (1.7)
AmpaAbena	28	2	30 (6.7)
Bonsaaso Pond	36	0	36 (0)
Bonsaaso River	21	0	21 (0)
Bowkrom	45	1	46 (2.2)
Nyame Bekyere	18	2	20 (10.0)
Pakro	17	9	26 (34.6)
Subin	60	6	66 (9.1)
Subin River	43	3	46 (6.5)
Tontokrom	36	1	37 (2.7)
Watreso	37	0	37 (0)
DobloGonno	21	0	21 (0)
BU–endemic total	537	42	579 (6.3)
BU nonendemic			
Abbeypanya	49	6	55 (10.9)
Aduman	11	6	17 (35.3)
Adumanya	38	0	38 (0)
Adwumam	41	5	46 (10.9)
Afienya	76	2	78 (2.6)
Asebi	22	3	25 (12.0)
Ayikumen	20	0	20 (0)
Bretsekrom	47	1	48 (2.1)
Dodowa	17	1	18 (5.6)
Keedmos	20	1	21 (4.8)
Odumse	47	4	51 (7.8)
Weija	36	0	36 (0)
BU–nonendemic total	424	29	453 (7.7)

*ER, enoyl-reduction-domain; BU, buruli ulcer. Communities are grouped as BU endemic or BU nonendemic based on BU cases during 2003–2005. Positiv percentages are given for each group as well as for the overall total. *t* test showed no significant difference between BU–endemic and BU–nonendemic site (t = 0.378, df = 25, p = 0.709).

Table 4. Taxon-specific ER PCR sample numbers and positivity results*

Higher classification	Lower classification	Family	BU endemic (N = 15) No. ER positive	BU endemic (N = 15) No. samples tested (% ER positive)	BU nonendemic (N = 12) No. ER positive	BU nonendemic (N = 12) No. samples tested (% ER positive)	All sites No. ER positive	All sites No. samples tested (% ER positive)
Bivalvia		Bivalvia				2 (0)		2 (0)
		Sphaeriidae			1	1 (100.0)	1	1 (100.0)
Gastropoda		Ancylidae		1 (0)		3 (0)		4 (0)
		Lymnaeidae		2 (0)				2 (0)
		Physidae	1	7 (14.3)		2 (0)	1	9 (11.1)
		Pilidae		5 (0)				5 (0)
		Planorbidae	1	18 (5.6)	1	11 (9.1)	2	29 (6.9)
		Bulininae	1	8 (12.5)	0	2 (0)	1	10 (10.0)
		Thiaridae		4 (0)		4 (0)		8 (0)
		Viviparidae		2 (0)		1 (0)		3 (0)
		Unknown		1 (0)		8 (0)		9 (0)
Nematoda				2 (0)		1 (0)		3 (0)
Oligochaeta			1	13 (7.7)	1	12 (8.3)	2	25 (8.0)
Hirudinea			2	15 (13.3)	1	10 (10.0)	3	25 (12.0)
Crustacea	Cladocera			5 (0)		8 (0)		13 (0)
	Mysidacea					1 (0)		1 (0)
	Conchostraca			1 (0)		0		1 (0)
	Copepoda			5 (0)		8 (0)		13 (0)
	Ostracoda			4 (0)	2	10 (20.0)	2	14 (14.3)
	Decapoda	Atyidae		4 (0)		12 (0)		16 (0)
Arachnida	Acari		3	15 (20.0)		13 (0)	3	28 (10.7)
Insecta	Collembola	Entomobryidae		3 (0)		3 (0)		6 (0)
		Isotomidae		1 (0)				1 (0)
	Ephemeroptera	Baetidae	2	21 (9.5)		23 (0)	2	44 (4.5)
		Caenidae	2	12 (16.7)	1	16 (6.3)	3	28 (10.7)
		Heptageniidae		3 (0)		1 (0)		4 (0)
		Leptophlebiidae		3 (0)				3 (0)
		Polymitarcyidae				4 (0)		4 (0)
		Unknown		1 (0)				1 (0)
	Odonata	Aeshnidae		1 (0)		2 (0)		3 (0)
		Anisoptera		7 0.0		10 (0)		17 (0)
		Calopterygidae				1 (0)		1 (0)
		Coenagrionidae		12 (0)		7 (0)		19 (0)
		Corduliidae		3 (0)		2 (0)		5 (0)
		Gomphidae				1 (0)		1 (0)
		Libellulidae	1	16 (6.3)	3	10 (30.0)	4	26 (15.4)
		Protoneuridae	3	11 (27.3)	1	13 (7.7)	4	24 (16.7)
		Zygoptera		5 (0)		8 (0)		13 (0)
	Hemiptera	Belostomatidae	2	21 (9.5)	2	16 (12.5)	4	37 (10.8)
		Corixidae		4 (0)		11 (0)		15 (0)
		Gerridae		8 (0)		9 (0)		17 (0)
		Hebridae		2 (0)				2 (0)
		Hydrometridae		5 (0)		6 (0)		11 (0)
		Mesoveliidae		17 (0)		17 (0)		34 (0)

		Naucoridae	2	12 (16.7)	1	6 (16.7)	3	18 (16.7)
		Nepidae	1	9 (11.1)	3	12 (25.0)	4	21 (19.0)
		Notonectidae	1	18 (5.6)	2	19 (10.5)	3	37 (8.1)
		Pleidae		15 (0)		9 (0)		24 (0)
		Saldidae		1 (0)		1 (0)		2 (0)
		Veliidae		8 (0)		6 (0)		14 (0)
	Lepidoptera	Pyralidae	2	3 (66.7)			2	3 (66.7)
	Coleoptera	Curculionidae				1 (0)		1 (0)
		Dytiscidae	1	27 (3.7)	2	17 (11.8)	3	44 (6.8)
		Elmidae		3 (0)	2	2 (100.0)	2	5 (40.0)
		Gyrinidae		2 (0)				2 (0)
		Hydraenidae	1	9 (11.1)	1	5 (20.0)	2	14 (14.3)
		Hydrophilidae	4	36 (11.1)	1	22 (4.5)	5	58 (8.6)
		Hygrobiidae		1 (0)				1 (0)
		Lampyridae		3 (0)		2 (0)		5 (0)
		Noteridae	1	23 (4.3)		15 (0)	1	38 (2.6)
		Psephenidae		1 (0)				1 (0)
		Scirtidae	1	6 (16.7)		4 (0)	1	10 (10.0)
		Unknown				7 (0)		7 (0)
	Trichoptera	Helicopsychidae				1 (0)		1 (0)
		Hydropsychidae		2 (0)				2 (0)
		Leptoceridae		4 (0)		2 (0)		6 (0)
		Polycentropedidae		1 (0)				1 (0)
	Diptera	Ceratopogonidae		15 (0)		13 (0)		28 (0)
		Chaoboridae		4 (0)		3 (0)		7 (0)
		Chironomidae	2	30 (6.7)	2	27 (7.4)	4	57 (7.0)
		Culicidae	2	19 (10.5)		11 (0)	2	30 (6.7)
		Dolichopodidae				1 (0)		1 (0)
		Ephydridae		1 (0)				1 (0)
		Psychodidae	1	1 (100.0)			1	1 (100.0)
		Sciomyzidae	1	1 (100.0)		2 (0)	1	3 (33.3)
		Stratiomyidae		5 (0)		2 (0)		7 (0)
		Syrphidae	1	1 (100.0)		1 (0)	1	2 (50.0)
		Tabanidae		3 (0)				3 (0)
		Tipulidae		4 (0)		2 (0)		6 (0)
		Unknown				2 (0)		2 (0)
Vertebrata	Anura		2	21 (9.5)	2	10 (20.0)	4	31 (12.9)
		Total	42	557 (7.5)	29	474 (6.1)	71	1,031 (6.9)
		Mean	1.6	8.0 (8.8)	1.5	7.2 (6.5)	2.4	12.7 (7.8)
		SD	0.8	8.0 (21.7)	0.8	6.3 (18.2)	1.2	13.8 (18.6)

*ER, enoyl-reduction-domain; BU, Buruli ulcer. Results are grouped as endemic or nonendemic on the basis of BU cases during 2003–2005. Positivity percentages are given for each group as well as for the overall total. The table also includes the total number of endemic or nonendemic sites where there was at least one ER postive for the taxon.
